# A rare case of metaplastic breast carcinoma from India: Towards precision oncology

**DOI:** 10.1002/cnr2.1997

**Published:** 2024-02-29

**Authors:** Soirindhri Banerjee, Ishika Mahajan, Aruni Ghose, Stergios Boussios, Shivam Chakraborty

**Affiliations:** ^1^ Department of Radiation Oncology Institute of Post Graduate Medical Education & Research and SSKM Hospital Kolkata India; ^2^ Department of Haematology and Oncology Lincoln County Hospital, United Lincolnshire Hospitals Trust Lincoln UK; ^3^ Department of Medical Oncology Barts Cancer Centre, St. Bartholomew's Hospital, Barts Health NHS Trust London UK; ^4^ Department of Medical Oncology Mount Vernon Cancer Centre, East and North Hertfordshire NHS Trust London UK; ^5^ Department of Medical Oncology Medway NHS Foundation Trust Kent UK; ^6^ United Kingdom and Ireland Global Cancer Network London UK; ^7^ Immuno‐Oncology Clinical Network Kent UK; ^8^ Faculty of Life Sciences and Medicine School of Cancer and Pharmaceutical Sciences, King's College London London UK; ^9^ Kent and Medway Medical School University of Kent Canterbury UK; ^10^ AELIA Organisation Thessaloniki Greece; ^11^ Depatment of Pathology Institute of Post Graduate Medical Education & Research and SSKM Hospital Kolkata India

**Keywords:** adenosquamoid differentiation, adjuvant radiotherapy, breast cancer, metaplastic, targeted therapy, triple negative

## Abstract

**Background:**

Metaplastic Breast Cancer (MpBC) is an exceedingly rare entity, accounting for less than 1% of all malignant breast tumours. Predominantly triple‐negative, they are notorious for their chemoresistance, high rates of recurrence and decreased disease‐free survival (DFS). All this contributes significantly to BC mortality and results in poor prognostic implications. Limited evidence has led to a lacuna of specific treatment guidelines for this entity and hence remains an uncharted territory for clinicians.

**Case:**

We report a case of a 46 year old premenopausal female with left‐sided metaplastic triple negative T3N2aM0 BC with mesenchymal differentiation (high grade) whom we treated with neoadjuvant chemotherapy, primary surgery in the form of extreme oncoplasty and adjuvant radiotherapy by Telecobalt machine. Contrary to the expected aggressive course of the disease and poor prognosis of treatment, the patient is presently in remission without progression for over 2 years of follow up.

**Conclusion:**

Limited experience in management of this pathological entity warrants the need for more research on it, with a special focus on targeted therapy. Discussing possibilities of a tailored approach, rather than a one‐size‐fits‐all approach may aid in paving the path for the future of MpBC treatment.

## 1 INTRODUCTION

A rare entity constituting 0.2%–5% of the global breast cancer (BC) burden, metaplastic breast cancer (MpBC)^1^ first described in the early 1980s represents a significant proportion of global BC mortality. They are mostly high‐grade tumours, demonstrating at least two unique cellular types—epithelial and mesenchymal elements mixed with carcinoma of the usual kind.^2^ These metaplastic changes represent a conversion from glandular breast tissue to non‐glandular carcinomatous (squamous) and sarcomatous (spindle cell, chondroid, osseous and rhabdoid cells) morphologies.^3^

The high‐grade variants like metaplastic variants have a high likelihood of metastasis and are notoriously chemoresistant and aggressive.^4^ The rarer low‐grade variants have a relatively favourable prognosis as compared to the commoner high‐grade subtypes.^5^

These tumours are typically triple‐negative,^3^, ^4^ distinct from conventional TNBC in proteomics and genomics. Spindle‐cell carcinoma commonly expresses p63 and low‐grade adenosquamous carcinoma show high rates of PIK3CA. Conventional TNBC have low PIK3CA expression. Osteoid and chondroid variants show increased SNail, BCL‐2‐like protein and Akt‐1 pathway activity. In contrast to conventional TNBC tumours, MpBC show higher upregulation of epithelial‐to‐mesenchymal transition (EMT) and collagen genes, but downregulation of keratinization genes. These support the hypothesis that the histological, proteomic and genomic variations may contribute to the aggressiveness of these BC variants^6^ resulting in shorter disease‐free interval and overall survival with a double chance of recurrence.^7^

We herein report a case of metaplastic carcinoma of the breast with mesenchymal differentiation (MCMD) in a premenopausal mother of two, conventionally treated as per BC guidelines, currently in her 3rd year of follow up without progression. This is a unique scenario given the fact that MCMD is documented to be a very aggressive tumour that has been recently classified as a subtype of metaplastic breast carcinoma, previously known as carcinosarcoma. Accounting for only 0.08%–0.2% of all breast cancers with only a few cases reported in literature, MCMD is characterised by a biphasic pattern of malignant epithelial and sarcomatous components without evidence of a transition zone between the two elements.^8^ They are characterised by larger size, lower rates of axillary node involvement, higher rates of triple negativity and distal metastases, earlier local recurrence and poorer survival, as compared with classic invasive breast cancer.

Surgery and radiotherapy remain the prime curative modalities for MpBC, in general, since these tumours have shown suboptimal response to standard chemotherapy. Such patients may be appropriate candidates for exploring novel targeted therapies. Owing to the scanty incidence of MpBC, adequate data on treatment outcomes has not been documented yet.

## 2 CASE

A 46‐year‐old premenopausal, diabetic lady with two living children presented to the outpatient clinic of the Radiation Oncology department of SSKM Hospital, Kolkata in September 2020 with a left‐sided breast lump.

On clinical examination, the lump measured 5 × 6 cm, occupying the upper half of the left breast. It extended to the nipple‐areolar complex with no fixity to the skin or underlying tissue. There were no skin changes. Axillary palpation showed multiple left‐sided matted lymph nodes.

Elaboration of a risk factor history revealed a 5‐year history of oral contraceptive pills usage 20 years previously.

An Ultrasonogram (USG) of bilateral breasts and axillae showed a large hypoechoic space‐occupying lesion, measuring about 6.5 × 4.6 cm, with irregular, mildly lobulated margins on the left upper breast at 12 o'clock position. The lesion showed no calcification or necrosis. Two oval lymph nodes measuring 1.2 and 1.4 cm in the largest diameter with noted in the left axilla. The scan was classified as BIRADS‐4.

A trucut biopsy was done shortly after the presentation. The histopathology of the sample was suggestive of invasive BC. Immunohistochemistry (IHC) revealed hormone receptors (oestrogen and progesterone) and HER2‐neu negative‐ triple negative breast cancer (TNBC). Fine needle aspiration cytology from the axillary lymph nodes was done, which revealed malignant ductal cells, suggesting metastatic involvement.

Oncological work‐up including a chest x‐ray (CXR) and USG of her abdomen revealed no abnormalities. A Tc99 m bone scan was advised, but the patient was unable to get it done due to economic constraints.

The tumour was staged at T3N2aM0 and the patient was discussed in a multidisciplinary team meeting. She was planned for surgical clip placement to delineate the tumour margins followed by neoadjuvant chemotherapy (NACT), re‐imaging and definitive surgery. A further plan was to be made following the histopathological examination of the surgical specimen.

She underwent 6 cycles of NACT with intravenous docetaxel (75 mg/m^2^), doxorubicin (50 mg/m^2^) and cyclophosphamide (500 mg/m^2^) at 3 weekly intervals. Chemotherapy was tolerated well.

**FIGURE 1:**
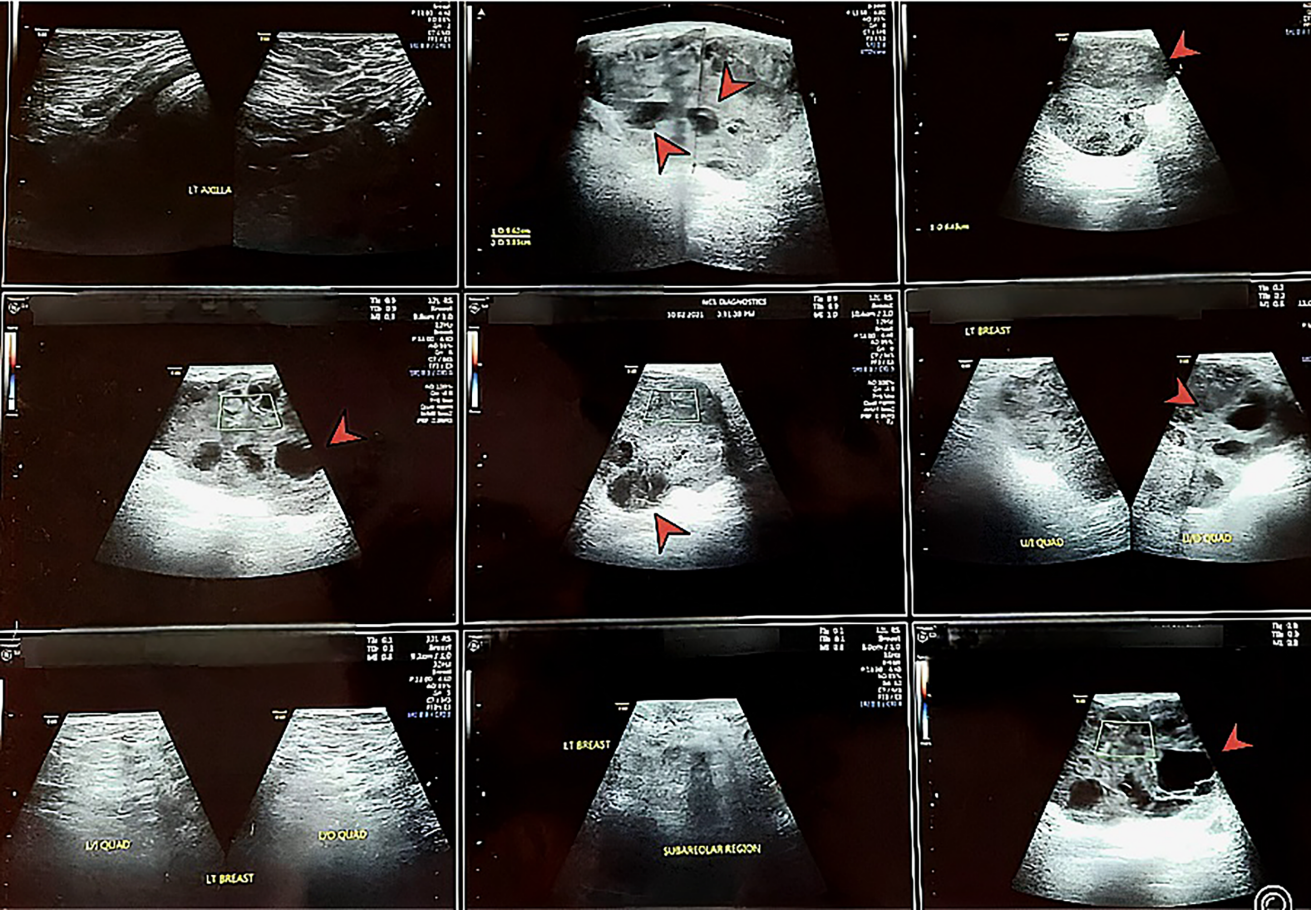
Interval USG of bilateral breasts and axillae done post 6 cycles of NACT (February 2021) showing left breast SOL (indicated by red arrows) measuring 9.6 × 6.4 × 5.9 cm in the upper quadrant with a BIRADS score of 6.

An interval USG of both breasts was done post‐chemotherapy, which showed an interval increase in the size of the lesion‐ 9.4 × 6.4 × 5.9 cm. There were internal echoes noted, likely necrotic foci within the lesion, with no significant axillary adenopathy. The right breast remained normal. The scan was classed BIRADS‐6 (Figure 1).

Following this, the option of surgery was discussed with the patient. She was keen on breast conservation. Due to a high breast: tumour ratio, she underwent a left‐sided extreme oncoplasty where the tumour and left axillary nodal tissue were removed en‐mass and reconstruction was performed with a latisimus dorsi musculocutaneous flap. The nipple‐areola complex was spared. The post‐operative period was uneventful.

**FIGURE 2:**
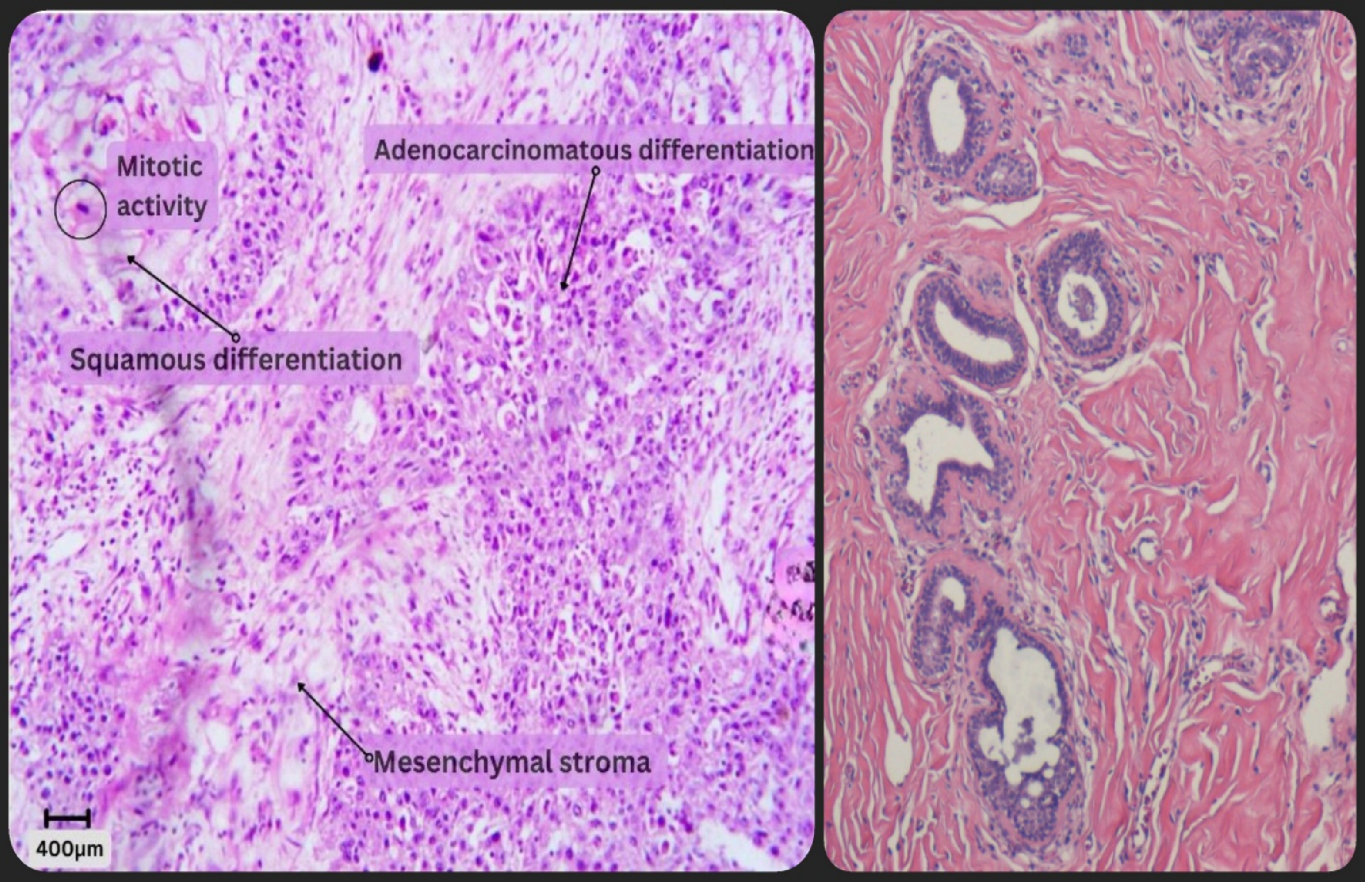
Left: Photomicrograph of the surgical specimen magnified to 100× (scaled to 1centimetre = 400 micrometres), showing haematoxylin & eosin stained nests, cords and acini of pleomorphic hyperchromatic squamoid cells alongside adenocarcinomatous differentiation in a background of spindle cell (mesenchymal) stroma, showing brisk mitotic activity; Right: Normal breast histology with acinar cells (myoepithelial) arranged in the form of large lobules interspersed by interlobular stroma.

Histopathological sections of the 18 × 13 × 6 cm surgical specimen showed a tumour measuring 7 × 6 × 4 cm. The tumour was composed of tubules, clusters, solid nests and syncytial cell infiltrate. (Figure 2) A large necrotic focus was also identified. There were areas of mesenchymal differentiation with pleomorphic cells and intervening occasional spindle cells. The cells had a variegated appearance and showed pleomorphism, prominent nucleoli and brisk mitosis. There was no evidence of lymphovascular or perineural invasion and no component of ductal carcinoma in situ. All resection margins and deep margins were clear. All resected axillary lymph nodes were negative. The tumour was classified as Grade 3, staged pT3N0Mx.

**FIGURE 3:**
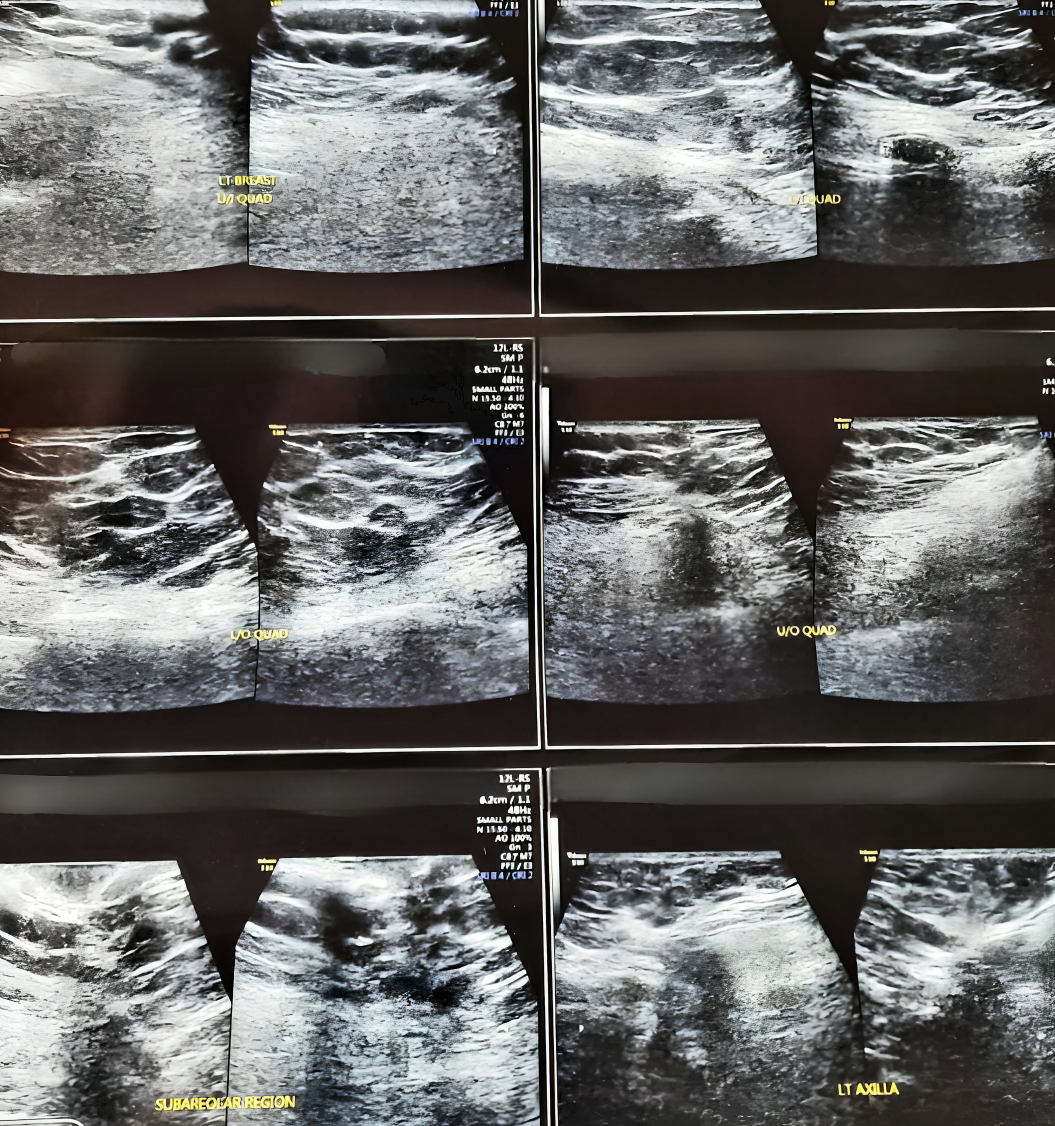
Follow‐up USG of bilateral breasts and axillae from December 2022 with normal parenchymal echogenicity, retromammary muscles and fat planes and no enlarged axillary lymph node suggestive of no recurrence in left breast or axilla.

She was rediscussed in the multidisciplinary team meeting. The meeting outcome was to treat her with adjuvant external beam radiotherapy (EBRT) targeting the chest wall and ipsilateral axillary lymph nodes with a Telecobalt‐60 machine (42.6 Grey in 16 fractions) with photon boost to the surgical bed, marked by the initial clips placed (10 Grey in 5 fractions) using right and left tangential fields. In June 2021, she completed EBRT uneventfully. Since then, she has been on 3 monthly follow‐up visits with USG of bilateral breast and axillae (Figure 3), serum CA15‐3 level and clinical breast examination alongside CT scan of thorax done 6 monthly. None of the aforesaid modalities have shown evidence of residual or recurrent disease and she has had no fresh complaints for the 2 years of follow up. There have been no treatment related complications so far and sequential subjective assessments during her follow‐up visits indicate that the patient's pre‐disease quality of life has been restored, in terms of cosmetic and psychosocial challenges and sexual and physiological activities of daily living.

## 3 DISCUSSION

Metaplastic breast cancer is an infrequent cancer of the breast, the identification, elucidation and management of which is an evolving field, gaining momentum over the last two decades.

The median age of presentation is 48–59 years, that is, perimenopausal women.^9^ Earlier database analyses have shown a higher mean age of diagnosis of 61 years.^10^ Our patient did not fit this demographic, with a younger age of presentation and premenopausal status. A higher prevalence is noted in African‐American and Hispanic women.^10^ There is a need to extend the databases to include Asian and African populations to identify risk groups in low‐middle‐income countries.

Clinically, the majority present with a large, well‐circumscribed mass, usually >5 cm.^11^ MpBC tends to have a large tumour size, rapid growth and less axillary lymph node involvement.^6^, ^12^ The present case had a similar large size at presentation keeping with the literature.

The diagnosis of MpBC is histopathological, thus is highly dependent on postoperative pathology. There is no typical imaging to discern it from the other variants of BC, and pathologically, as it is a mixture of two or more homologous or heterologous components, it can be very difficult to differentiate it from other rare benign or malignant histologies.^13^

Metaplastic carcinomas are on the spectrum of basal carcinomas, displaying a basal/myoepithelial and epithelial‐to‐mesenchymal molecular structure.^14^ It is a rare heterogenous subtype characterised by squamous, spindle cell and mesenchymal phenotype with or without conventional adenocarcinoma component.^15^

Histopathological categorisation is of cardinal importance as it guides the prognosis with the squamous variant being the worst. Diagnosis from cytology is challenging as both epithelial and mesenchymal elements are essential components. They are known to display positivity for cytokeratin, S‐100 and vimentin or myoepithelial markers like CD10, p63, and smooth muscle actin. These tumours are mostly sporadic but can arise from previous lesions like fibroadenoma, spindle cell carcinoma, papilloma and complex sclerosing lesions.^16^ Beatty et al. identified 24 MpBC cases, which showed high‐nuclear grade, negative ER/PR and HER2 status, epidermal growth factor receptor (EGFR) positivity and no significant difference in multidisciplinary treatment patterns, recurrence, or survival, in comparison to typical BC.^17^ Prior studies have found that MpBC typically has molecular alterations in epithelial‐to‐mesenchymal transition; amplification of EGFR/HER1; PI3K/AKT, nitric oxide and Wnt/β‐catenin signalling; altered immune response; and cell cycle dysregulation.^6^

In the present case, it was challenging to morphologically differentiate adenosquamous variant of metaplastic breast carcinoma from mesenchymal variant owing to prominent glandular and squamoid areas interspersed with mesenchymal areas. However, it was histologically more consistent with adenosquamous differentiation with spindle cell stroma along with high‐grade anaplasia. Further immunophenotyping could not be done at our institute at that time due to unavailable reagents for squamous/myoepithelial markers and neither could the patient afford the test from outside.

Most MpBC tumours are triple‐negative, and thus the management principles follow those of conventional TNBC. These cancers are treated with anthracycline, taxane and platinum‐based chemotherapy. The larger size of the tumours, lack of hormone therapy as a systemic treatment, and the increased risk of metastasis make a case for the increased use of systemic chemotherapy though the literature bases lack substantial evidence to support this practice.^5^, ^10^ Our patient received NACT, following which the axillary nodes did shrink (negative axillary dissection specimen), but the tumour however grew in size. The cut section did however demonstrate a large area of necrosis, which was pre‐empted by the interval sonomammogrphy showing internal echoes. Variation in response to NACT exists based on the histologic subtype, with some benefit observed in matrix‐producing MpBC.^9^ The role of NACT in MpBC is still unclear, but may continue to remain the standard of practice due to the higher risk of metastasis in the absence of it, and until newer novel therapies are developed. Taxane‐anthracycline‐platin regimen was selected for NACT of this patient, keeping in mind the potential aggressiveness of a metaplastic TNBC and good general condition of the patient without any concerning co‐morbidities. Dose‐dense regimens were avoided due to high‐patient burden at our institute, leading to logistic constraints.

There have been limited studies regarding the use of adjuvant radiotherapy, most of which have demonstrated better overall survival (OS), DFS and reduced recurrence rate.^18^ Following the conventional principles of BC treatment, radiation to the tumour bed is commonly given with BCS, which has shown some favourable outcomes. Unfortunately, the published literature has small patient cohorts.^19^

Most patients with MpBc receive surgery as a viable treatment option, especially if presented early with locally advanced operable tumours. Both mastectomy and breast conservation surgery were performed, with the former being more commonly performed due to larger tumour size, and high tumour:normal breast tissue ratio.^10^, ^20^

Novel molecular targeted therapies, such as poly ADP‐ribose polymerase (PARP) inhibitors, angiogenesis inhibitors (bevacizumab), protein kinase inhibitors and mammalian target of Rapamycin (mTOR) inhibitors (temsirolimus or everolimus) have shown good potential for research in MpBC. The increased expression of EGFR provides an opportunity for targeted tumour therapy in these tumours.^13^

Predictors of a poorer outcome are the presence of skin invasion, younger age at presentation (<39 years), and appearance of squamous cell carcinoma in the lymph nodes.^11^, ^16^

## 4 CONCLUSION

MpBC represents a heterogeneity in breast malignancies, with a need for tailoring treatment for the different variants of breast cancer, rather than approaching it as a single entity. Though triple negative adenosquamous MpBC is expected to have rapid progression to a metastatic disease with poor OS and DFS rates, the rare case reported here suggests that not all cases of high‐grade MpBC have a poor outcome with conventional therapeutic interventions and questions whether MpBC needs to be mandatorily labelled as a therapeutic challenge for both the oncologist and the patient. However, the scarcity of reported cases and lack of clear guidelines for management warrants the need for more research on the entity, with a special focus on targeted therapy for a tailored approach, rather than a one‐size‐fits‐all approach.

## AUTHOR CONTRIBUTIONS

**Soirindhri Banerjee:** Conceptualization (lead); formal analysis (lead); investigation (equal); resources (lead); validation (equal); writing – original draft (lead); writing – review and editing (lead). **Ishika Mahajan:** Methodology (lead); resources (supporting); validation (lead); writing – review and editing (lead). **Aruni Ghose:** Project administration (lead); supervision (lead). **Stergios Boussios:** Supervision (supporting). **Shivam Chakraborty:** Investigation (equal); resources (supporting); validation (equal); writing – original draft (supporting); writing – review and editing (supporting).

## FUNDING INFORMATION

None.

## CONFLICT OF INTEREST STATEMENT

The authors have stated explicitly that there are no conflicts of interest in connection with this article.

## ETHICS STATEMENT

Informed consent of the patient was obtained for publishing details of her disease and treatment.

## Data Availability

This is a case report of a single patient, whose details were recorded and reported. Identification data have not been disclosed to respect the privacy of the patient.
